# A Striational Muscle Antigen and Myasthenia Gravis-Associated Thymomas Share an Acetylcholine-Receptor Epitope

**DOI:** 10.1155/1992/86853

**Published:** 1992

**Authors:** Alexander Marx, Mary Osborn, Socrates Tzartos, Kerstin I. Geuder, Berthold Schalke, Wilfried Nix, Thomas Kirchner, Hans-Konrad MüLler-Hermelink

**Affiliations:** 1 lnstitute of Pathology University of Wüzburg Wüzburg 8700 Germany; 2 Pathologisches Institut der Universität Josef Schneider Str. 2 Würzburg 8700 Germany; 3 Max Planck Institute for Biophysical Chemistry Göttingen Germany; 4 Helenic Pasteur Institute Athens Germany; 5 Department of Neurology University of Waüzburg, Germany; 6 Department of Neurology University of Mainz Germany

**Keywords:** Thymoma, thymus, myasthenia gravis, acetylcholine receptor, titin, autoimmunity

## Abstract

The coincidence of autoantibodies against the acetylcholine receptor (AChR) and muscle striational antigens (SA) is a characteristic finding in thymoma-associated myasthenia gravis (MG), but their origins are still unresolved. Some common muscle antigens that were shown to be targets of anti-SA autoantibodies in thymoma-associated MG have also been detected in normal or neoplastic thymic epithelial cells, suggesting that the release of (eventually altered) antigens from the thymic tumors could elicit SA autoimmunity. In contrast to this model, we report here that titin, which is a recently reported target of SA autoimmunity, is not expressed in thymomas. In addition, we show that skeletal muscle type-II fibers exhibit a striational immunoreactivity with monoclonal antibody mAb155, which was previously identified to label a very immunogenic cytoplasmic epitope of the AChR and neoplastic epithelial cells of MGassociated thymomas. We conclude from these findings that titin autoimmunity in thymoma-associated MG is either due to a molecular mimicry mechanism involving tumor antigens (other than titin) or is a secondary phenomenon following release of titin from muscle. Based on the common immunoreactivity of the AChR, a striational antigen and thymoma, we suggest as the pathogenetic mechanism of thymoma-associated MGa "circulus vitiosus" in which SA autoimmunity could help maintain the AChR autoimmunity that is primarily elicited by the thymomas.


*Developmental Immunology*, 1992, Vol. 2, pp. 77–84
Reprints available directly from the publisher
Photocopying permitted by license only

© 1992 Harwood Academic Publishers GmbH
Printed in the United Kingdom

### A Striational Muscle Antigen and Myasthenia Gravis-Associated Thymomas Share an Acetylcholine-Receptor Epitope

ALEXANDER MARX,*† MARY OSBORN,‡ SOCRATES TZARTOS,§ KERSTIN I. GEUDER,† BERTHOLD SCHALKE,| | WILFRIED NIX,# THOMAS KIRCHNER,† and HANS-KONRAD MÜLLER-HERMELINK†

*†Institute of Pathology, University of Würzburg, 8700 Würzburg, Germany*
*‡Max Planck Institute for Biophysical Chemistry, Göttingen, Germany,*
*§Helenic Pasteur Institute, Athens, Greece*
*| | Department of Neurology, University of Würzburg, Germany*
*#Department of Neurology, University of Mainz, Germany*

The coincidence of autoantibodies against the acetylcholine receptor (AChR) and muscle striational antigens (SA) is a characteristic finding in thymoma-associated myasthenia gravis (MG), but their origins are still unresolved. Some common muscle antigens that were shown to be targets of anti-SA autoantibodies in thymoma-associated MG have also been detected in normal or neoplastic thymic epithelial cells, suggesting that the release of (eventually altered) antigens from the thymic tumors could elicit SA autoimmunity. In contrast to this model, we report here that titin, which is a recently reported target of SA autoimmunity, is not expressed in thymomas. In addition, we show that skeletal muscle type-II fibers exhibit a striational immunoreactivity with monoclonal antibody mAb155, which was previously identified to label a very immunogenic cytoplasmic epitope of the AChR and neoplastic epithelial cells of MG-associated thymomas. We conclude from these findings that titin autoimmunity in thymoma-associated MG is either due to a molecular mimicry mechanism involving tumor antigens (other than titin) or is a secondary phenomenon following release of titin from muscle. Based on the common immunoreactivity of the AChR, a striational antigen and thymoma, we suggest as the pathogenetic mechanism of thymoma-associated MGa "circulus vitiosus" in which SA autoimmunity could help maintain the AChR autoimmunity that is primarily elicited by the thymomas.

KEYWORDS: Thymoma, thymus, myasthenia gravis, acetylcholine receptor, titin, autoimmunity.

### INTRODUCTION

Myasthenia gravis (MG) is characterized by an abnormal fatiguability of muscle produced in most patients by autoantibodies against the nicotinic acetylcholine receptor (AChR). These autoantibodies are thought to impair neuromuscular transmission by reducing the number of muscle endplate AChR or blocking their function (Drachman et al., 1980; Burges et al., 1990).

In addition to anti-AChR autoimmunity, 70% of MG patients exhibit thymitis with or without lymphofollicular hyperplasia (Müller-Hermelink et al., 1986; Kirchner et al., 1986) and 10% have a thymoma (Kirchner and Müller-Hermelink, 1989). As an important diagnostic feature, only the latter group of MG patients almost invariably has high titers of heterogeneous autoantibodies against striational muscle antigens (SA) in addition to anti-AChR autoantibodies (Peers et al., 1977; Aarli et al., 1990; Connor et al., 1990; Ohta et al., 1990). In thymoma patients without MG anti-SA autoantibodies occur in only 24% (Williams and Lennon, 1987).

How SA autoimmunity is elicited in thymomas is not known. However, the finding that myosin and some less well-defined muscle proteins are anti-SA-autoantibody targets that also occur in

*Corresponding author: Present address: Pathologisches Institut der Universität, Josef Schneider Str. 2, 8700 Würzburg, Germany.

normal or neoplastic thymic epithelial cells (Gilhus et al., 1984; Williams and Lennon, 1987; Dardenne et al., 1987) has led to the hypothesis that thymomas might be the sites of SA autosensitization. Because an antigenic relationship between striational antigens and the AChR was not known, it is obscure whether autoimmune reactions against SA and AChR are interrelated.

Recently, titin was identified as a major target of anti-SA autoantibodies in MG (Aarli et al., 1990). In the present paper, we report that titin is not expressed in thymomas, suggesting that this kind of SA autoimmunity is either not due to intratumorous autosensitization or occurs by molecular mimicry mechanisms, that is, by titin-related antigenic determinants in thymoma proteins that may cross-react with titin-"specific" T cells, which could induce an antititin autoantibody response when released to the periphery. In addition, an immunoreactivity is demonstrated that is shared by the AChR alpha subunit and a striational antigen different from titin. Because the respective epitope—corresponding to AChR alpha371–378—is the only one so far detected in MG-associated thymomas, we suggest that AChR autoimmunity and part of SA autoimmunity in MG-associated thymomas could be interrelated.

### RESULTS

### Titin Is Expressed in Thymic Myoid Cells but Not in Thymomas

Frozen sections of two normal thymuses were probed with antititin mAb to three different titin epitopes. As shown in Fig. 1, immunoreactivity with all three mAbs was seen in a few round or spindle-shaped cells inside the medulla of normal thymuses close to Hassal's corpuscles. Both the localization and the morphology of these cells are typical of thymic myoid cells (Kirchner et al., 1988). This was confirmed by double immunolabeling of thymic myoid cells by both antidesmin or anti-AChR mAb and by antititin mAbT12 (Figs. 2a and 2b). There was no expression of titin in keratin-positive nonneopalstic thymic epithelial cells (Fig. 2c).

Because of the high incidence of antititin autoantibodies typical of MG/thymoma patients (Aarli et al., 1990), we looked for an aberrant expression of titin in thymoma because the denovo expression of other proteins has previously been reported as a typical feature of neoplastic thymic epithelium (Willcox et al., 1987; Marx et al., 1990, 1991). However, with respect to the

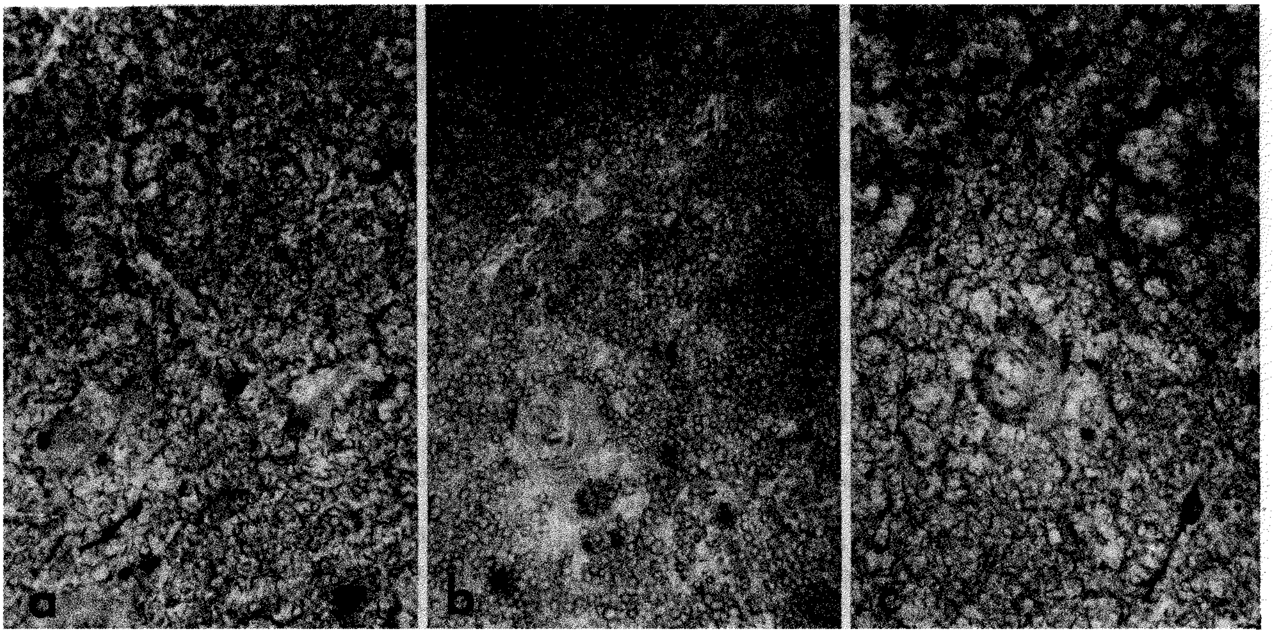

FIGURE 1. Immunoreactivity of a few round or spindle-shaped cells inside the human thymic medulla with antititin monoclonal antibodies T4 (a), T12 (b), and T32 (c). Morphology and localization are typical of thymic myoid cells (cf. Fig. 2). No immunoreactivity is encountered in epithelial cells. Immunoperoxidase technique on frozen sections (×160).

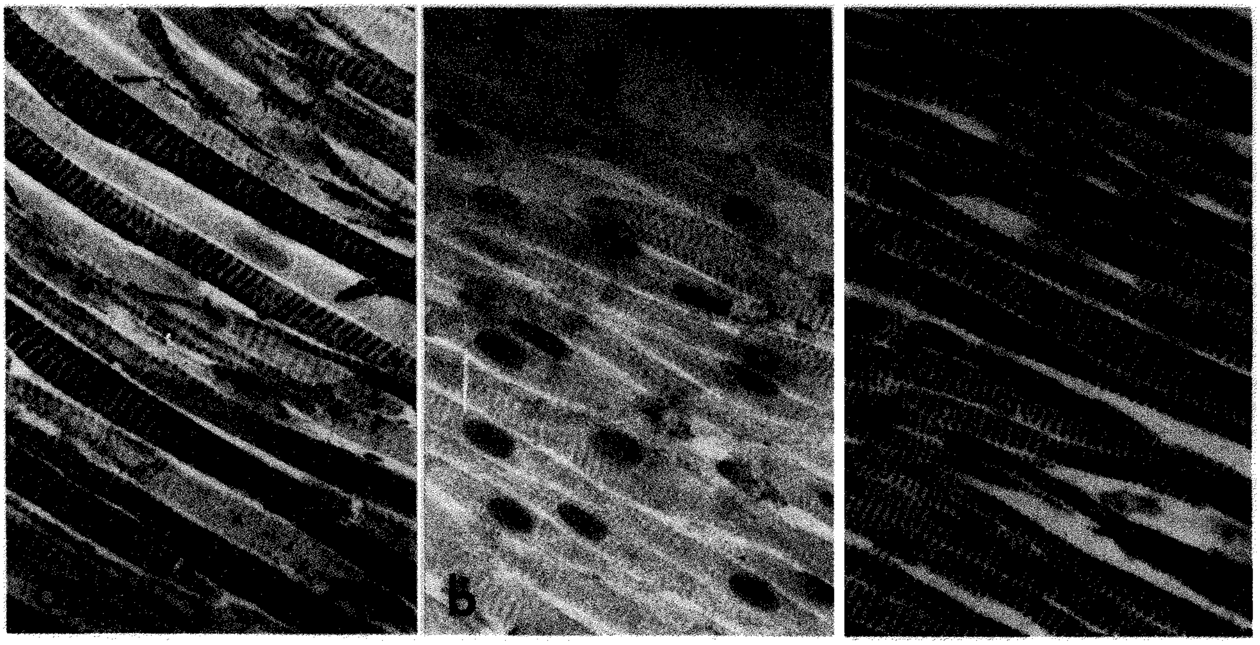

FIGURE 4. Immunoreactivity of a striational muscle antigen in about 50% of quadriceps muscle fibers with anti-AChR mAb155 directed against the AChR epitope alpha371–378 (a). No reactivity of muscle with anti-AChR mAb195 directed against the AChR "main immunogenic region" (Tzartos et al., 1983) (b). In contrast to the striational immunoreactivity of mAb155, the immunoreactivity with antititin mAb T12 is encountered in 100% of muscle fibers (c), demonstrating the nonidentity of titin and the AChR-epitope-bearing striational antigen in human quadriceps muscle (all sections #S361). Frozen sections, immunoperoxidase (×400).

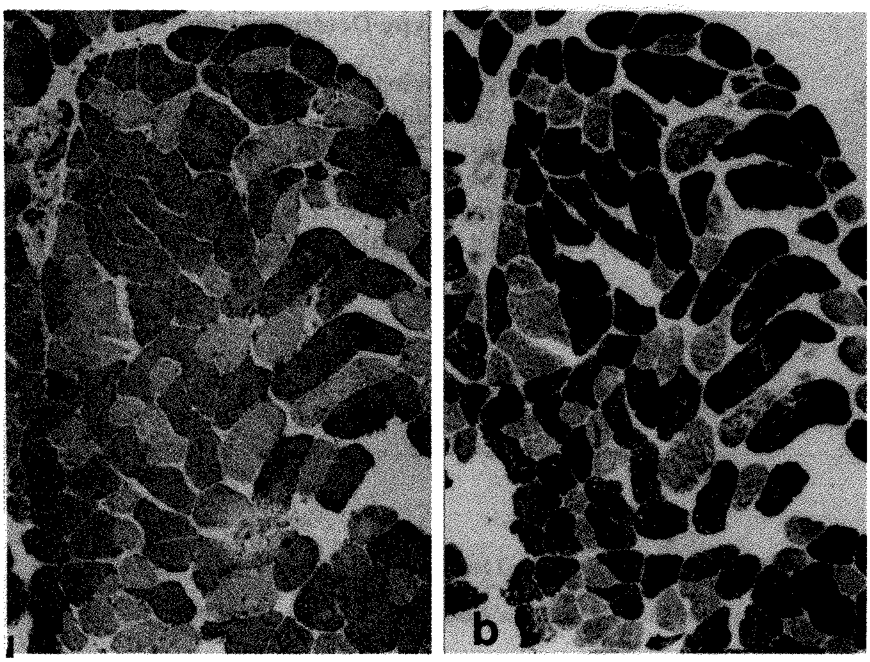

FIGURE 5. Immunoreactivity of anti-AChR mAb155 with a striational antigen is confined to type-II fibers in the quadriceps muscle. Serial cross sections labeled with mAb155 (a; immunoperoxidase) and reacted for ATPase activity at pH 9.4 (b). Immunoreactivity with mAb155 coincides with a high ATPase activity (dark color) typical of type-II fibers (Dubowitz, 1985) (× 400).

have to prove whether the striational antigen immunolabeled by mAb155 (Fig. 4a) is identical to the thymoma proteins bearing the AChR epitope detected by mAb 155 (Marx et al., 1989,

TABLE 1
Clinical and Pathological Findings in MG Patients with Thymic Epithelial Tumors and in Controls

| Case No. | Sex | Age (y) | Histologic diagnosis | AChR Epitope[a] |
|---|---|---|---|---|
| 19964 | F | 34 | Mixed thymoma | – |
| 29117 | F | 64 | Mixed thymoma | – |
| 15977 | F | 76 | Cortical thymoma | + |
| 6942 | M | 50 | Cortical thymoma | + |
| 22401 | F | 63 | Well-differentiated thymic carcinoma | + |
| 27920 | M | 45 | Well-differentiated thymic carcinoma | + |
| Thy2 | F | 2 | Normal thymus | +[b] |
| Thy40 | M | 40 | Normal thymus | +[b] |
| S361 | M | 2 | Quadriceps muscle | +[c] |
| S64 | F | 81 | Quadriceps muscle | +[c] |

[a]Immunoreactivity with anti-AChR mAb155 (Tzartos et al., 1986).
[b]Immunoreactivity of mAb155 with thymic myoid cells and some medullary epithelial cells (Kirchner and Müller-Hermelink, 1989).
[c]Striational pattern in type-II fibers.

1990), and whether either molecule is in fact a target of autoimmune B or T cells *in vivo*. If the latter question could be positively answered, our finding suggests a pathogenetic model in which striational autoimmunity might help to maintain AChR autoimmunity. Such a model of a "circulus vitiosus" would explain the clinical experience that removal of the thymoma does not ameliorate the MG.

### MATERIALS AND METHODS

### Patients and Tissues

Six thymomas from MG patients, two normal thymuses (obtained during thoracic surgery), and normal quadriceps muscle (obtained from autopsies within 6 hr after death) were investigated using cryostat sections from snap frozen tissue. Tumors were classified according to Kirchner and Müller-Hermelink (1990). Clinical data of patients are summarized in Table 1.

The diagnosis of generalized myasthenia gravis was based on clinical findings, including an abnormal muscle fatiguability, electrophysiological investigations and anti-AChR serum titers that were above 1 nmol/L.

### Immunohistochemistry

The monoclonal antibodies (mAb) used in this study are described in Table 2. The immunohistochemical three-stage immunoperoxidase technique and the double-labeling procedure combining the immunoperoxidase with an alkaline phosphatase technique (as the first and second step, respectively) were as described previously, including the same control experiments (Kirchner et al., 1988).

### Muscle Fiber-Type Determination

To investigate whether skeletal muscle immunoreactivity with anti-AChR mAb155 was confined to either type-I or -II muscle fibers serial frozen sections were processed for routine enzyme histochemical determination of ATPase activity at both pH 4.6 and pH 9.4 (Dubowitz, 1985).

TABLE 2
Antibodies Used in This Study

| Antibody | Concentration or dilution applied | Antigen labeled | Source |
|---|---|---|---|
| 35betaH11 | 1:100 | Keratin No. 8/18 | Gown and Vogel, 1982 |
| Desmin | 1:1000 | Desmin | Laboserv Giessen, Germany |
| T4 | 1:50 | Nonrepetitive titin epitope inside the I band | Fürst et al., 1988 |
| T12 | 1:10 | Nonrepetitive titin epitope close to the Z line | Fürst et al., 1988 |
| T32 | 1:5 | Repetitive titin epitope within the A band | Fürst et al., 1989 |
| mAb155 | 1 $\mu$g/mL | AChR-epitope alpha371–378 | Tzartos et al., 1986 |
| mAb195 | 1 $\mu$g/mL | AChR-epitope alpha67–76 (MIR[a]) | Tzartos et al., 1988 |

[a]MIR=main immunogenic region (Tzartos et al., 1988).

### ACKNOWLEDGMENTS

We thank Ms Ch. Kohaut and Ms A. Pietz and Mr E. Schmitt for expert technical assistance, and Professor W. Roggendorf for support concerning muscle fiber typing. Supported by grant Ki 370/1-1 of the German Research Foundation (DFG) to A.M. and T.K.

*(Received May 14, 1991)*

*(Accepted September 25, 1991)*

### REFERENCES

Aarli J.A., Stefansson K., Marton L.S.G., and Wollmann R.L. (1990). Patients with myasthenia gravis have in their sera IgG autoantibodies against titin. Clin. Exp. Immunol. **82:** 284–288.

Burges J., Wray D.W., Pizzighella S., Hall Z., and Vincent A. (1990). A myasthenia gravis plasma immunoglobulin reduces miniature endplate potentials at human endplates in vitro. Muscle-Nerve **13:** 407–413.

Connor R.I., Levfert A.K., Benes S.C., and Lang R.W. (1990). Incidence and reactivity pattern of skeletal and hert (SH) reactive autoantibodies in the sera of patients with myasthenia gravis. J. Neuroimmunol. **26:** 147–157.

Dardenne M., Savino W., and Bach J.F. (1987). Thymomatous epithelial cells and skeletal muscle share a common epitope by a monoclonal antibody. Am. J. Pathol. **126:** 194–198.

Drachman D.B., Adams R.N., Stanley E.F., and Pestronk A. (1980). Mechanisms of acetylcholine receptor loss in myasthenia gravis. J. Neurol. Neurosurg. Psychiatry **43:** 601–610.

Dubowitz V. (1985). Muscle Biopsy, 2d edition (London: Bailliere Tindall Inc.), pp. 63–67.

Fuji Y., Monden Y., Nakahara K., Hashimoto J., and Kawashima Y. (1984). Antibody to acetylcholine receptor in myasthenia gravis: Production by lymphocytes from thymus and thymoma. Neurology **34:** 1182–1186.

Fürst D.O., Nave R., Osborn M., and Weber K. (1989). Repetitive titin epitopes with a 42 nm spacing coincide with known A band striations also identified by major myosin-associated proteins. J. Cell Sci. **94:** 119–125.

Füst D.O., Osborn M., Nave R., and Weber K. (1988). The organization of titin filaments in the half-sarcomer revealed by monoclonal antibodies in immunoelectron microscopy: A map of ten nonrepetitive epitopes starting at the Z line extends close to the M line. J. Cell Biol. **106:** 1563–1572.

Geuder K.I., Schoepfer R., Kirchner T., Marx A., and Müller-Hermelink H.K. (1989). The gene of the alpha-subunit of the acetylcholine receptor: Molecular organization and transcription in myasthenia-associated thymomas. Thymus **14:** 179–186.

Gilhus N.E., Aarli J.A., Christensson B., and Matre R. (1984). Rabbit antiserum to citric acid extract of human skeletal muscle staining thymomas from myasthenia gravis patients. J. Neuroimmunol. **7:** 55–64.

Gown A.M., and Vogel A.M. (1982). Monoclonal antibodies to intermediate filament proteins of human cells: Unique and crossreacting antibodies. J. Cell Biol. **95:** 414–424.

Hohlfeld R. (1990). Editorial. Myasthenia gravis and thymoma: Paraneoplastic failure of neuromuscular transmission. Lab. Invest. **62:** 241–243.

Hohlfeld R., Toyka K.V., Miner L.L., Walgrave S.L., and Conti-Tronconi B. (1988). Amphipathic segment of the nicotinic receptor alpha-subunit contains epitopes recognized by T lymphocytes in myasthenia gravis. J. Clin. Invest. **81:** 657–660.

Kao I., and Drachman D.B. (1977). Thymus muscle cells bear acetylcholine receptors: Possible relation to myasthenia gravis. Science **195:** 74–75.

Kirchner T., Hoppe F., Schalke B., and Müller-Hermelink H.K. (1988b). Microenvironment of thymic myoid cells in myasthenia gravis. Virchows Arch (Cell Pathol.) **54:** 295–302.

Kirchner T., and Müller-Hermelink H.K. (1989). New approaches to the diagnosis of thymic epithelial tumors. *In:* Progress in Surgical Pathology, vol. 10, Fenoglio-Preiser C.M., Wolff M., Rilke F., Eds. pp. 167–186. (Philadelphia: Field and Wood Inc.)

Kirchner T., Schalke B., Melms A., Kügelgen T., and Müller-Hermelink H.K. (1986). Immunohistochemical patterns of non-neoplastic changes in the thymus in myasthenia gravis. Virchows Arch. (Cell Pathol.) **52:** 237–257.

Kirchner T., Tzartos S., Hoppe F., Schalke B., Wekerle H., and Müller-Hermelink H.K. (1988a). Pathogenesis of myasthenia gravis: Acetylcholine receptor-related antigenic determinants in tumor-free thymuses and thymic epithelial tumors. Am. J. Pathol. **130:** 268–280.

Marx A., Geuder K.I., Schoepfer R., Tzartos S., Kristoffersson U., Schalke B., Kirchner T., and Müller-Hermelink H.K. (1991). Analysis of the acetylcholine receptor epitope bearing protein p153 in thymomas favours "false-positive T cell selection" as a mechanism of paraneoplastic myasthenia gravis. *In:* Lymphatic tissues and immune responses, Imhof B., et al., Eds. (New York: Marcel Dekker), pp. 577–583.

Marx A., Kirchner T., Hoppe F., O'Connor R., Schalke B., Tzartos S., and Müller-Hermelink H.K. (1989). Proteins with epitopes of the acetylcholine receptor in epithelial cell cultures of thymomas in myasthenia gravis. Am. J. Pathol. **134:** 865–877.

Marx A., O'Connor R., Geuder K.I., Hoppe F., Schalke B., Tzartos S., Kalies I., Kirchner T., and Müller-Hermelink H.K. (1990). Characterization of a protein with an acetylcholine receptor epitope from myasthenia gravis-associated thymomas. Lab. Invest. **62:** 279–286.

Melms A., Chrestl S., Schalke B.C.G., Wekerle H., Mauron A., Ballivet M., and Barcas T. (1989). Autoimmune T lymphocytes in myasthenia gravis: Determination of target epitopes using T-lines and recombinants of the mouse nicotinic acetylcholine receptor gene. J. Clin. Invest. **83:** 785–790.

Müller-Hermelink H.K., Marino M., and Palestro G. (1986). The human thymus: Histophysiology and pathology. Current topics in pathology, vol. 75, Müller-Hermelink H.K., Ed. (Berlin: Springer).

Newsome-Davis J., Willcox N., Schluep M., Harcourt G., Vincent A., Mossman S., Wray D., and Burges J. (1987). Immunological heterogeneity and cellular mechanisms in myasthenia gravis. Ann. N.Y. Acad. Sci. **505:** 12–26.

Ohta M., Ohta K., Itoh N., Kurobe M., Hayashi K., and Nishitani H. (1990). Anti-skeletal muscle antibodies in the sera from myasthenia patients with thymoma: Identification of anti-myosin, actomysin, actin, and alpha-actinin antibodies by solid-phase radioimmunoassay and a Western blotting analysis. Clinica Chimica Act **187:** 255–264.

Peers J., McDonald B.L., and Dawkins R.L. (1977). The reactivity of the antistriational antibodies associated with thymoma and myasthenia gravis. Clin. Exp. Immunol. **27:** 66–73.

Schluep M., Willcox N., Vincent A., Dhoot G.K., and Newsom-Davis J. (1987). Acetylcholine receptors in human thymic myoid cells in situ: An immunohistological study. Ann. Neurol. **22:** 212–220.

Sommer N., Willcox N., Harcourt G.C., and Newsom-Davis J. (1990). Myasthenia thymus and thymoma are selectively enriched in AChR-reactive T cells. Ann. Neurol. **28:** 312–319.

Tzartos S.J., Kokla A., Walgrave S.L., and Conti-Tronconi B.M. (1988). Localization of the main immunogenic region of human acetylcholine receptor to residues 67–76 of the alpha subunit. Proc. Natl. Acad. Sci. USA **85:** 2899–2903.

Tzartos S., Langeberg L., Hochschwender S., Swanson L.W., and Lindstrom J. (1986). Characteristics of monoclonal antibodies to denatured Torpedo and to calf actylcholine receptors: Species, subunit and region specificity. J. Neuroimmunol. **10:** 235–253.

Vincent A., Whitting P., Schluep M., Heidenreich F., Lang B., Roberts A., Willcox N., and Newsom-Davis J. (1987). Antibody heterogeneity and specificity in myasthenia gravis. Ann. N.Y. Acad. Sci. **505:** 106–120.

Wekerle H., Ketelsen U.P., Zurn A.D. and Fulpius B.W. (1978). Intrathymic pathogenesis of myasthenia gravis: Transient expression of acetylcholine receptors on thymus derived myogenic cells. Eur. J. Immunol. **8:** 579–581.

Willcox N., Schluep M., Ritter M.A., Schuurman H.J., Newsom-Davis H., and Christensson B. (1987). Myasthenic and non-myasthenic thymoma. An expansion of a minor cortical epithelial cell subset. Am. J. Pathol. **127:** 447–460.

Williams C.L., and Lennon V.A. (1986). Thymic B-lymphocyte clones from patients with myasthenia gravis secrete monoclonal striational antibodies reacting with myosin, alpha-actinin, or actin. J. Exp. Med. **164:** 1043–1059.

